# Bio-Recognition in Spectroscopy-Based Biosensors for *Heavy Metals-Water and Waterborne Contamination Analysis

**DOI:** 10.3390/bios9030096

**Published:** 2019-07-30

**Authors:** Alessandra Aloisi, Antonio Della Torre, Angelantonio De Benedetto, Rosaria Rinaldi

**Affiliations:** 1 Institute for Microelectronics and Microsystems (IMM), CNR, Via Monteroni, 73100 Lecce, Italy; 2 Mathematics and Physics “E. De Giorgi” Department, University of Salento, Via Monteroni, 73100 Lecce, Italy; 3 ISUFI, University of Salento, Via Monteroni, 73100 Lecce, Italy

**Keywords:** water pollution, environmental water, drinking water, milk, heavy metal ions, biosensor, detection limits, optical spectroscopy, proteins, functional nucleic acids

## Abstract

Microsystems and biomolecules integration as well multiplexing determinations are key aspects of sensing devices in the field of heavy metal contamination monitoring. The present review collects the most relevant information about optical biosensors development in the last decade. Focus is put on analytical characteristics and applications that are dependent on: (i) Signal transduction method (luminescence, colorimetry, evanescent wave (EW), surface-enhanced Raman spectroscopy (SERS), Förster resonance energy transfer (FRET), surface plasmon resonance (SPR); (ii) biorecognition molecules employed (proteins, nucleic acids, aptamers, and enzymes). The biosensing systems applied (or applicable) to water and milk samples will be considered for a comparative analysis, with an emphasis on water as the primary source of possible contamination along the food chain.

## 1. Introduction

Biosensors are currently valid tools, other than laboratory analytical instrumentation, for monitoring the quality of natural water (e.g., in the food production chain) [1]. Biosensors are not meant to take over standard analytical methods, but, when optimal features of a sensing device are met, they offer remarkable advantages over conventional techniques. Overall, in certain conditions, their promptness and low-cost manufacturing make them useful tools to analyze many samples for primary warnings. As defined by the International Union of Pure and Applied Chemistry (IUPAC), “a biosensor is an integrated receptor ± transducer device, capable of providing selective analytical information using a biological recognition element” [2]. Optical biosensors are a group of sensors in which (i) the transducer senses optical fluctuations in the input light resultant from bioreceptor—target interaction, and (ii) the amplitude of these changes hinge on the concentration of the analyte [1].

Even in very small amounts, several metal ions may have important effects on health state, as they are hardly degradable but easily accumulated in the body through the diet [3]. Metal ions are generally not essential nutrients; conversely, they could be damaging to all living species [4].

* Widely indicated as “heavy metals” (HMs), in a technical report of 2002, the author concluded: “The term *heavy metal* has never been defined by any authoritative body such as IUPAC. No relationship can be found between density and any of the various physicochemical concepts that have been used to define *heavy metals* and the toxicity attributed to *heavy metals*… Understanding bioavailability is the key to assessment of the potential toxicity... It depends on biological parameters and on the physicochemical properties of metallic elements, their ions, and their compounds. These in turn depend upon the atomic structure of the metallic elements, systematically described by the periodic table” [5]. 

In the last twenty years, with the aim to quantify trace amounts of such possible contaminants, environmental monitoring has generated a need for innovative and improved approaches that have ever-increasing sensitivity and selectivity, as described in a recent review paper on various analytical techniques-based biosensors [6]. The introduction of biosensors has brought in new and promising approaches, but with still limited application in the environmental field if compared with the biomedical one, where most efforts have converged in the past years. 

Much research is still needed before biosensors consolidate as a recognized analytical strategy with respect to environmental and food trace contaminant detection. 

**Figure 1:**
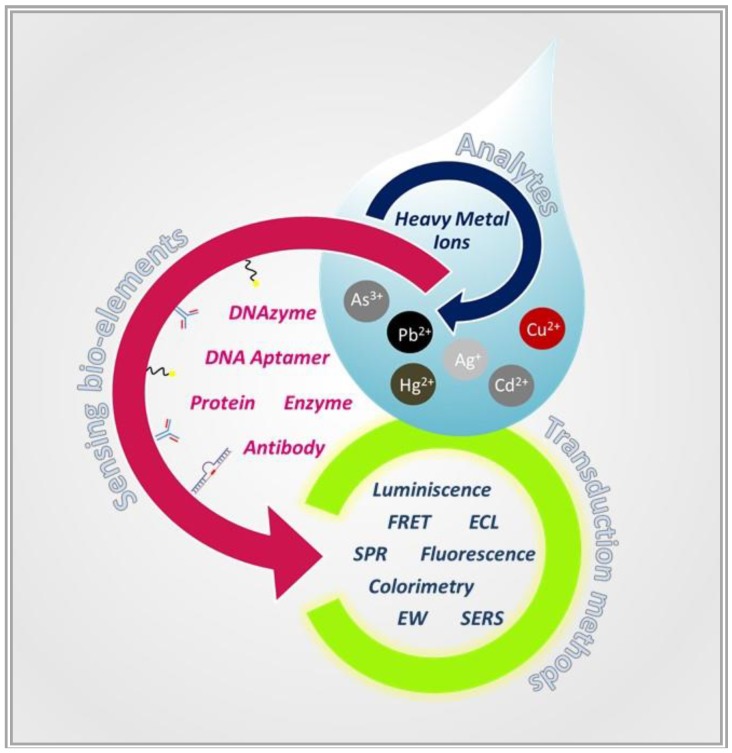
Optical biosensor scheme strategies for heavy metal (HM) ion detection in a water/milk “drop”. Transduction methods and bioreceptor classes synergistically employed for the development of recently published devices.

In this direction, the integration of nanomaterials and functional biological molecules is part of a new era in the optical biosensor area. Actually, nano-structured materials unveil distinctive size- and shape-dependent physicochemical properties, showing a number of possible interactions [7] with the biorecognition component, which may act as a reaction catalyst, or may be in equilibrium with macromolecules present in their natural biological settings or isolated and engineered [2]. Essentially, while the sensor sensitivity is influenced by the selected transducer component, the bioreceptor is responsible for the specificity [8]. Many biosensing elements that can be coupled to different transducers are now available for HM detection (Figure 1). 

In general, depending on the specific mechanism of the bioreceptor component, five groups can be identified: (i) DNA-based metal biosensor, (ii) antibodies, (iii) proteins, (iv) cellular structures or whole cells, (v) biomimetic receptors (gene-engineered molecules, molecularly imprinted polymers [9], and molecularly imprinted membranes [10], mimicking a natural bioreceptor. Most of them, natural and synthetic, are exhaustively described in recent review papers (concerning the interaction of metal ions with DNAs, peptides and enzymes, whole cells, as well as ionophores and small molecules) [8,11,12,13,14,15].

From a functional point of view, optical biosensors can be further categorized as: (i) probing biosensors: Entailing sensors based on target and recognition element affinity interaction; (ii) reacting biosensors: Where the optical responses relies on chemical processes [16]. Concerning the biomolecular probes, the most widely exploited can be collected into two macro groups: proteins and nucleic acids. The specific affinities of these two families of molecules for HM ions are briefly introduced below, before entering the focal topic of this paper.

With regard to metal binding proteins, phytochelatins or metallothionein, metal ligands found in plants, are usually exploited on the surface of the transducer, where protein–metal interactions occur through the formation of a complex [17,18]. Functional proteins with enzymatic activity (purified or directly in a microorganism) catalyze specific chemical reactions also in the presence of metal ions. The mechanisms of action of these elements embrace: (a) Transformation of the analyte into a sensor-detectable product, (b) detection of an analyte behaving as negative or positive enzyme activity modulator, or (iii) appraisal of enzyme properties deviations upon interaction with the analyte [19,20].

Metal ions affinity for amino acid side chains (with sulfur, nitrogen, and oxygen atoms) and the occurrence of such amino acids in antibody-determining regions are expected to influence the ability of antibodies to strongly bind to metal–chelate complexes [21,22,23,24].

On a parallel route, functional nucleic acids (FNAs) represent molecules whose usefulness is further than that of encoding genetic information [25], and whose chemical structure is suitable for metal recognition. Two active structures have been developed for this purpose, working as either direct metal binding or metal-assisted deoxyribonucleic/ribonucleic acid catalyst. Definitely, aptamers, metal ion-specific DNA, guanine (G)-rich oligonucleotides, and DNA-based enzymes (DNAzymes) are the most widely reported [26]. In brief, aptamers are able to effectively bind basically any molecule of choice; they consist of artificial short single-stranded (ss) nucleic acid sequences or peptide molecules identified by combinatorial selection, through the Systematic evolution of ligands by exponential enrichment (SELEX) methodology [27,28] that, upon binding to targets, can fold into specific secondary and tertiary structures [29,30].

Basically, DNA and metal ions may interact in three different ways: (i) By HM ions-based exchanging of hydrogen atoms of the Watson–Crick base pairs; (ii) by reversible binding of HM ions with DNA; (iii) forming kinetically inert complexes by persistent crosslinking of DNA with HM ions [31]. The metal ion-specific DNAs are those sequences most commonly rich in thymine (T) or cytosine (C), with great selectivity for metal ions, which promote robust metal-base complex formation—specifically forming T–Hg^2+^–T [32] and C–Ag^+^–C mismatch [33]. G-rich DNAs are G-rich strands with a tendency to self-associate into non-canonical secondary structures named G-quadruplexes (G4) [34]. On these cations coordination-induced structure/property changes, a number of strategies have been proposed for the detection of Pb^2+^, Hg^2+^, Ba^2+^, Ag^2+^, K^+^ [26]. 

A different class is then represented by nucleic acid enzymes (Ribozymes and DNAzymes). These are molecules found in nature like catalytic RNA or in vitro selected DNA sequences, displaying specific strong metal-dependent activity and structure recognition capability, bypassing the need for metal immobilization [25,26]. 

Remarkably, the choice of a suitable biological element and transduction module makes the biosensor sensitive and analyte specific, thus efficient for toxicological studies. Portable biosensors also make in-situ analysis possible, facilitating real-time monitoring [35].

In this context, the present work aims to review the sensitivity of HM-dedicated optical biosensor systems published in the last decade. Several biosensors relevant for water sample or liquid food monitoring are here described, although only those showing HM ion detection in real and complex matrices are compared, as reported in Table 1. 

All methods are listed in order of the prevalence of published biosensors for HM sensing in water or milk matrix, be it a real or laboratory-built aqueous sample. Analytical techniques here presented include luminescence, colorimetry, evanescent wave, surface-enhanced Raman spectroscopy, Förster resonance energy transfer, and surface plasmon resonance. As the core purpose of this review is to recognize which method displays the maximum stated sensitivity—for the selected HM ion, focusing on the biosensing element employed—additional focused tables (Table 2 and Table 3) have been worked out and introduced later in the text.

## 2. Biosensing Methods

**Figure 2:**
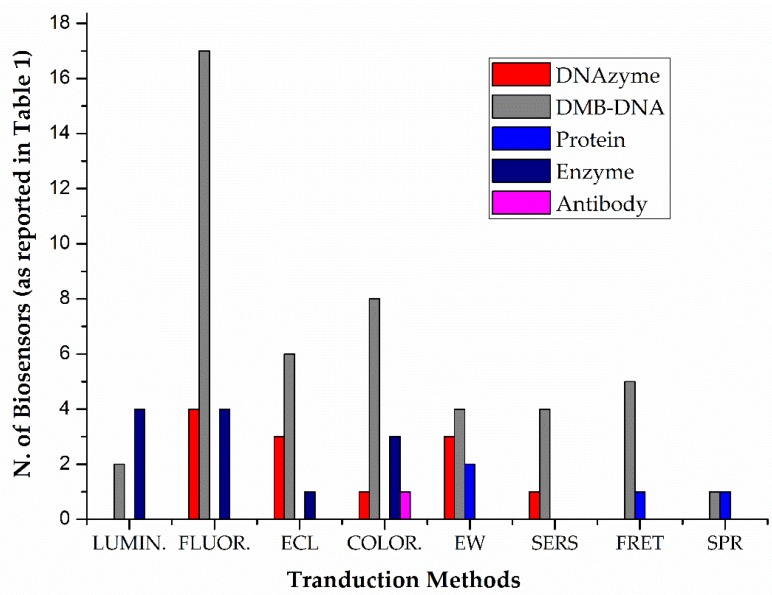
Distribution of biorecognition elements exploited in recently reported sensors for HM detection in real samples, as classified in Table 1.

In order to introduce a brief summary of what the reader will encounter during this paragraph, in Figure 2, the HM ion optical biosensor distribution is plotted with respect to the recognition element used, in the frame of the same transduction method, as already classified in Table 1.

With regards to the already mentioned classes of molecules, some considerations have emerged: (1) FNAs are the most employed; (2) direct metal binding DNA sequences (DMB-DNA) subclass, comprising aptamer, metal ion-specific DNA, and G-rich oligonucleotide, occupies a wide portion in the described FNAs-operating sensors; (3) proteins are the least employed, and (4) catalytic active protein-based sensors have been proposed more than those exploiting a non-catalytic protein, or a specific antibody. 

**Figure 3:**
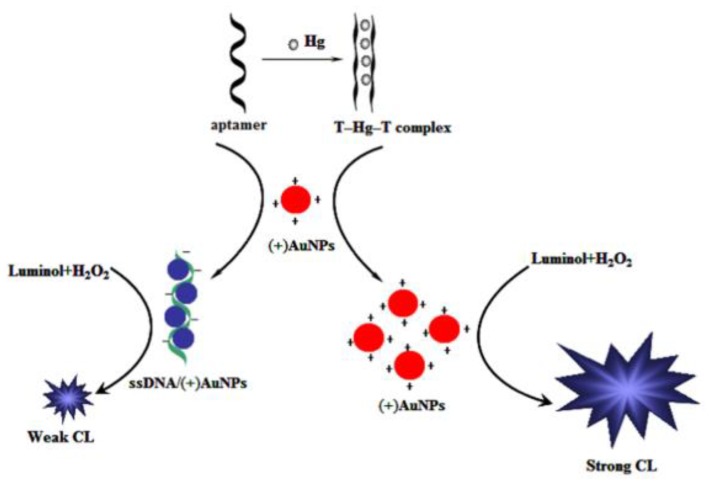
Metal ion-induced T–T complex mechanism for CL-based HM detection. Hg^2+^ induces T–Hg^2+^–T complex formation preventing the interaction between aptamer and positive AuNPs, allowing the catalytic reaction occurrence and a stronger CL signal emission. From ref. [38], with the permission of the Publisher.

**Figure 4:**
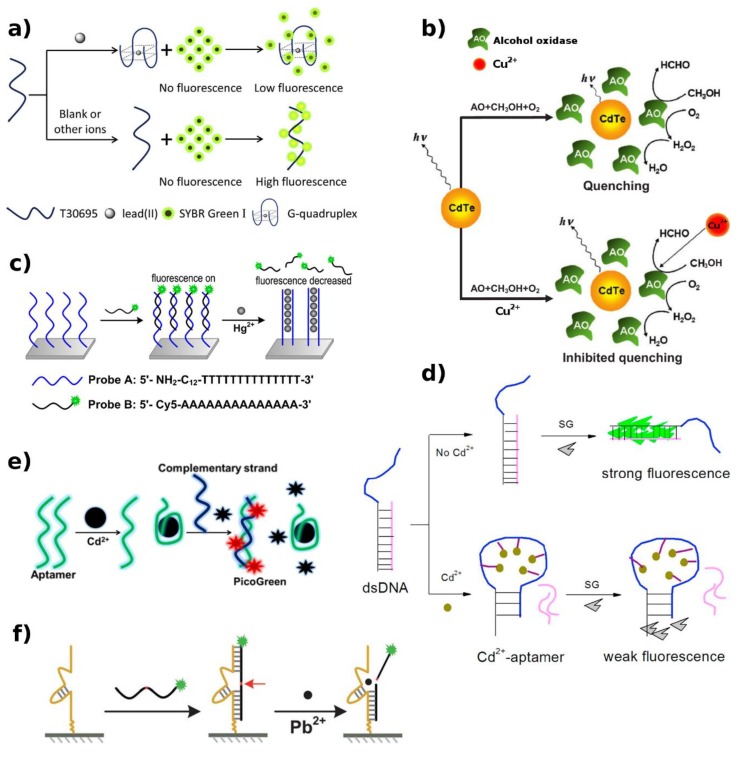
Various biosensing element constructs for fluorescence-based HM detection. (**a**) Pb^2+^-stabilized G4 formation for turn off detection. In the presence of Pb^2+^, the T30695 oligonucleotide folds into a G4 structure, leading to a PL signal reduction [64]. (**b**) Cu^2+^-determined enzymatic inhibition for turn on detection. AO catalyzes the oxidation of methanol to hydrogen peroxide, inducing the quenching of QDs fluorescence. Cu^2+^ ions inhibits the enzymatic activity decreasing the quenching of QDs fluorescence [48]. (**c**) Metallophilic attraction of the Hg atom in the T–Hg^2+^–T base pair mismatch. The Hg^2+^-induced dislocation of the complementary labeled poly–A strand allows the turn-off detection mechanism. [51]. (**d**) Cd^2+^-induced hairpin formation. The release of the complementary strand from dsDNA and the sequence conformational switching to a stem-loop structure lead to a fluorescence decay of the signal reporter [73]. (**e**) Random coil structure to aptamer–Cd^2+^ complex. After the addition of complementary strands and PG, the residual free aptamer that did not bind with Cd^2+^ forms a hybrid with complementary strands and PG dye which results in a big fluorescent enhancement [72]. (**f**) Pb^2+^-induced hydrolytic cleavage signal-off. The catalytic strand carries out catalytic reactions for hydrolytic scission of the substrate sequence at the rA site (red arrow). Once the substrate is broken into two pieces, it dissociates from the catalytic strand with a decrease of the surface PL intensities [54]. Adapted with the permission of the Publishers.

**Figure 5:**
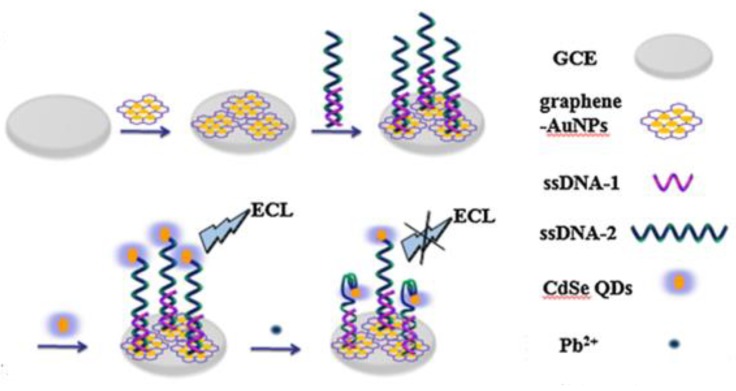
Metal ion-induced quadruplex construct for ECL-based HM detection The Pb^2+^ causes the G4 structure formation of the G-rich ssDNA, leading to a shortening of the distance between the CdSe QDs and the graphene–AuNPs nanocomposite, thus inducing a reduction of the ECL signal. From [84], with the permission of the Publisher.

**Figure 6:**
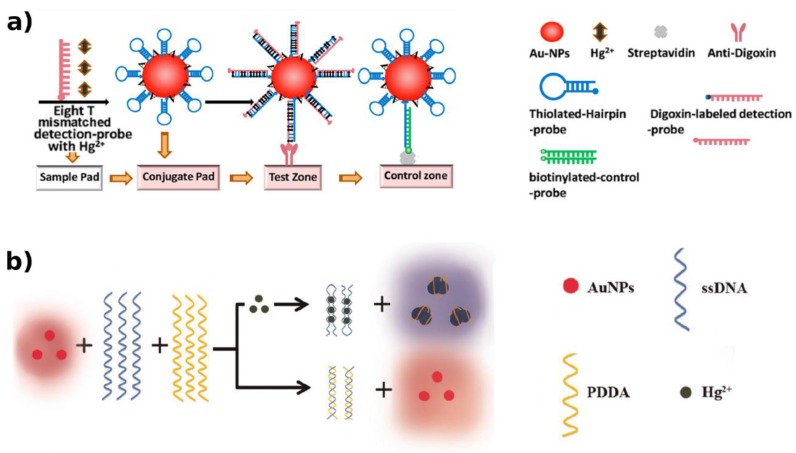
T–Hg^2+^–T structure and hairpin probe-modified AuNPs-based strategy for colorimetry- based HM detection. (**a**) The digoxin dsDNA–AuNPs complexes are captured by immunoreaction with the anti-digoxin Ab* immobilized on the strip and revealed by a red band [98]. (**b**) With the formation of the T–Hg^2+^–T, a random coil-to-hairpin structure change occurs, avoiding ssDNA interaction with PDDA. A color change is observed due to the AuNP aggregation by free PDDA [89]. Adapted with the permission of the Publishers.

**Figure 7:**
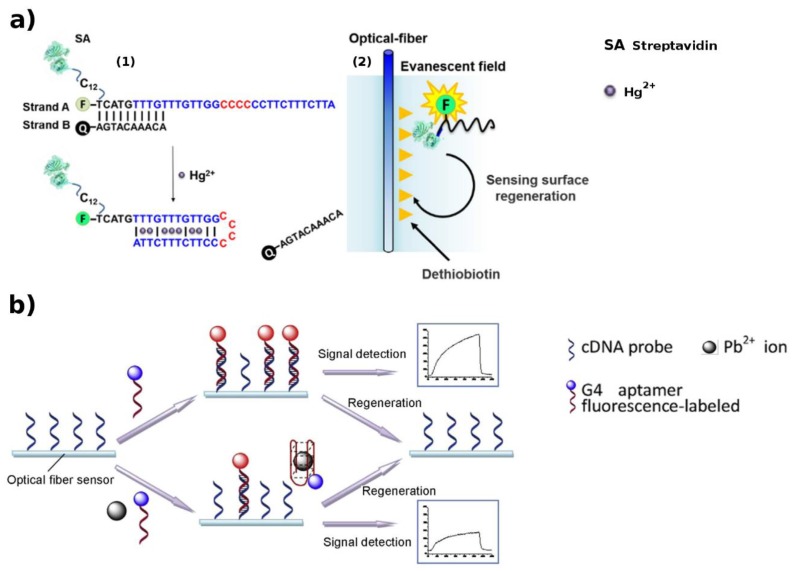
FNAs constructs for evanescent wave-based HM detection (**a**) (1) T–T mismatch-driven biosensing by triple functional DNA–protein conjugates for facile detection of mercury ions; (2) Once the DNA–SA is bound to form a hairpin structure stabilized by the T–T mismatch, an enhancement in the signal is observed [105]. (**b**) G4-driven lead ions biosensing. A decrease of fluorescence signal is recorded by the Pb^2+^-induced aptamer conformation change (G4) that reduces the binding of the fluorescently-labeled free DNA with the immobilized complementary strand [108]. Adapted with the permission of the Publishers.

**Figure 8:**
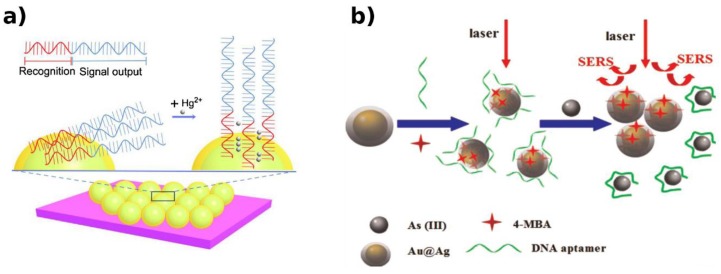
FNAs constructs for SERS-based HM detection. (**a**) Hg^2+^ causes a vertical alignment of DNA molecules due to the formation of T–Hg^2+^–T pairs via N–Hg^2+^–N J-coupling bonding, changing respective Raman intensities [113]. (**b**) As^3+^ induces the aptamer release from NP surface, inducing NP aggregation and the generation of SERS “hot spots” [112]. Adapted with permission of the Publishers.

**Figure 9:**
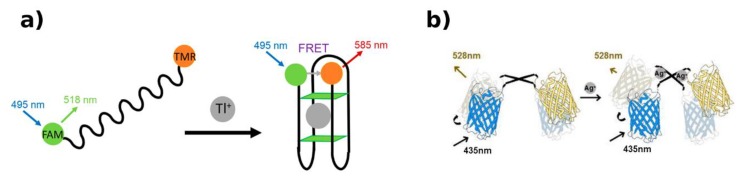
FNAs and protein constructs for FRET-based HM detection. (**a**) The Tl^+^ causes the G4 structure formation, leading to a shortening of the distance between the donor and acceptor, thus inducing an enhancement in FRET efficiency [88]. (**b**) The decrease in FRET efficiency is induced by conformational change of the CupR protein, in the presence of Ag^+^ [121]. Adapted with permission of the Publishers.

**Figure 10:**
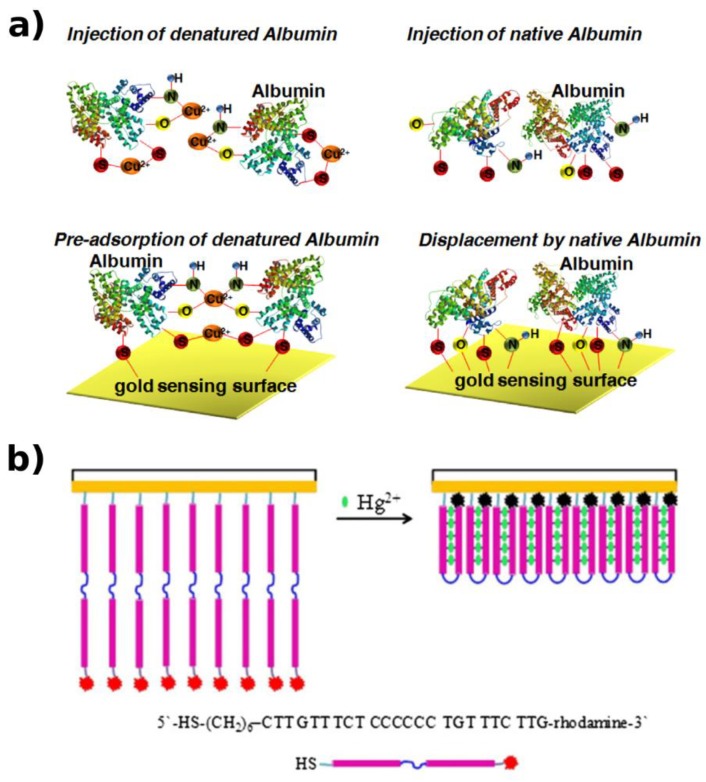
(**a**) Protein and DNA structure conformational changes, for SPR-based HM detection. The interaction between native proteins and Cu^2+^ leads to the protein structure denaturation and weakens its attraction on the sensing surface. The competitive displacement by the native one causes variations in the SPR angle profile [126]. (**b**) The rhodamine-labeled ssDNA folds into the T–Hg^2+^–T-mediated hairpin loop; this structural change approaches the rhodamine fraction near to the Au surface causing the increase in the SPR signal and the PL quenching [127]. Adapted with permission of the Publishers.

In the next subparagraphs, the newly developed biosensors based on these recognition elements will be described, and with regard to the exploited biosensing mechanism, the more representative strategies will be showed in summary figures (Figure 3, Figure 4, Figure 5, Figure 6, Figure 7, Figure 8, Figure 9 and Figure 10).

### 2.1. Luminescence

Luminescence concerns the emission of light from an excited electronic state of an atom or molecular species. A luminescence phenomenon that occurs when a chemical reaction triggers the excitation of an electronic state in a molecular species, that decays emitting light, is named chemiluminescence (CL) [36]; luminescence caused by electrogenerated chemical excitation is named electrochemiluminescence (ECL) [37]. Another luminescence phenomenon is photoluminescence (PL), where a molecule absorbs light, and then decays to a lower energy excited electronic state emitting light with a wavelength different than that of the absorbed light. Depending on the average lifetime of the excited state, the luminescence band can either be fluorescence or phosphorescence [36]. 

A number of biosensors exploit these phenomena and are here reported. A CL aptasensor for Hg^2+^ detection, with a limit of detection (LOD) of 16 pM, was designed by Qi et al. [38]. The sensor is based on positively-charged gold nanoparticles (AuNPs) effect, that show catalytic properties for CL reaction of luminol and H_2_O_2_, and on aptamer conformation change induced by Hg^2+^. In the absence of Hg^2+^, the aptamer causes a weak CL signal because it wraps on positive AuNPs reducing their catalytic properties. Whereas the presence of Hg^2+^ leads to a T–Hg^2+^–T complex formation preventing the interaction between aptamer and positive AuNPs, allowing the catalytic reaction to occur (Figure 3).

A different Hg^2+^ biosensor, based on two label-free DNA probes and the molecular light switch complex ${\left[Ru{\left(phen\right)}_{2}\left(dppz\right)\right]}^{2+}$, was developed by X. Zhang et al. [39]. If Hg^2+^ is present, the two label-free DNA probes, with eight T–T mismatches, form stable DNA duplexes which allow the intercalation of ${\left[Ru{\left(phen\right)}_{2}\left(dppz\right)\right]}^{2+}$, leading to a significant Hg^2+^-dependent enhancement of the luminescence intensity. A LOD of 3.5 × 10^−10^ M was reached. 

A portable multianalyte device, based on a different recognition strategy was designed by R. K. Mishra et al. for Hg^2+^, Pb^2+^, and Cd^2+^ [40], obtaining a LOD of 1, 0.7, and 0.02 μg/L respectively. The device exploits a luminol–H_2_O_2_ mixture as a chemiluminescent system and horseradish peroxidase (HRP). The enzymatic inhibition results in a CL suppression that is analyte concentration dependent. Though, in a previous work, Deshpande et al. [41], exploiting a two enzyme based (i.e., alcohol oxidase (AlOx) and HRP) inhibition assay for single HM ion determination, showed a lower LOD (1 pg/mL) for Hg^2+^ ions.

Recently, semiconductor sensors have received significant consideration. Electrochemically-etched nano-porous silicon (PS) is considered as a promising material for luminescent chemical sensors [42,43]. Interestingly, PS layers were exploited to develop novel enzyme-based biosensor systems for determination of glucose and urea (direct) as well as HM ions (inhibitory) [44]. In particular, changes in the quantum yield of PS photoluminescence at variations in medium pH. In particular, changes in the quantum yield of PS photoluminescence at variation in medium pH is proposed for the biosensor system. The authors show that the presence of Cu^2+^, Pb^2+^, or Cd^2+^ ions causes an inhibition of the enzymatic reactions, resulting in a restoration of the PL quantum yield of PS. The LOD of the biosensor was approximately 10 nM. In order to develop handheld enzymatic luminescent biosensors for HMs detection, the integration of luciferase-based microfluidic chip with a portable luminometer has been also realized [45]. The LOD reached for Cu^2+^ sulfate was 2.5 mg/L.

#### 2.1.1. Fluorescence

Transducing the molecular recognition events with the fluorescence signals is very attractive and is one of the most widely adopted methods [46]. Simultaneous measurements of multi-elements were arranged by an array-based biosensor exploiting enzymatic activity [47]. Acetylcholinesterase and urease were exploited as model enzymes and combined with a sensing probe (FITC–dextran), for the assessment of pH, urea, acetylcholine, and HMs. A LOD lowered to 10 nM was achieved for Hg^2+^ and a LOD of 50 µM was reported for Cd^2+^.

A different kind of fluorescent transducer successfully constructed for determination of Cu^2+^ in surface water, exploits the combination of semiconductor quantum dots (QDs) and enzymatic inhibition [48]. AlOx catalyzes methanol oxidation to produce H_2_O_2_, inducing the quenching of QDs fluorescence. Copper ions inhibit the enzyme action and, consequently, the quenching of QDs fluorescence decreases (Figure 4b). This hybrid sensor showed a LOD of 2.75 nM. 

Useful as new fluorescent sensors, carbon-based QDs (CQDs, namely biodots) have attracted growing interests thanks to their biocompatibility, chemical inertness, and water solubility. In this direction, an application of DNA-derived CQDs in metal ion sensing was demonstrated [49]. Hg^2+^ and Ag^+^ are predisposed to be captured by the DNA biodots due to the existence of T and C groups (leading to T–Hg^2+^–T or C–Ag^+^–C complex), resulting in a quenched fluorescence, with the largest efficiency obtained at pH 7 and a LOD of 48 nM for Hg^2+^ and 0.31 µM^.^ for Ag^+^.

A turn-on aptasensor for Hg^2+^ detection based on graphene oxide (GO) and DNA aptamers was proposed, where GO plays a role as nano quencher (Q) to reduce the fluorescence of acridine orange (AO). The recognition process results in the simultaneous formation of T–Hg^2+^–T and G4 structures; the formed G4 can capture AO from the GO surface, leading to fluorescence retrieval. A LOD of 0.17 nM was achieved [50].

Similarly, based on the T–Hg^2+^–T coordination between two neighboring poly–T strands, two ready-to-use chip-based sensors match well with microarray technology for Hg^2+^ detection in the turn-on and turn-off modality [51]. The induced dislocation of the complementary poly-adenine (poly–A) strand, labeled with either a fluorophore (F) or a (Q), allows the turn-off and turn-on detection of Hg^2+^, respectively (Figure 4c). A lower LOD was achieved in the turn-off mode (3.6 vs. 8.6 nM). 

Remarkably, with the aim to remove the HM-fluorescence quenching effect, a magnetic separation was integrated for Hg^2+^ sensing based on the formation of the T–Hg^2+^–T structure [52], allowing a LOD value of 0.2 nM.

Another multi-analyte biosensor based on parallel analysis of microarray technology was developed exploiting DNAzymes [53]. In particular, copper and lead ion-dependent DNAzymes are first associated with their corresponding DNA substrates on the surface of aldehyde-modified slides. Then, in the presence of the specific ions, the DNA cleavage of the substrate takes place, inducing a strong variation in fluorescence signal. The sensor showed a LOD value of 0.6 ppb for Cu^2+^ and 2 ppb for Pb^2+^. A higher sensitivity for Pb^2+^, with a LOD of 1 nM, was achieved by a similar approach, exploiting a Cy5-labeled DNA/RNA chimera (Figure 4f) as substrate [54]. 

Working on complex real samples, enzymatic degradation represents a threat to the structural integrity of D-DNAzymes. In this context, L-DNAzymes show similar recognition capability and catalytic capacity with respect to their enantiomer. A promising biosensor for Pb^2+^ ion detection was realized by building a Pb^2+^-specific L-DNAzyme, allowing to obtain a LOD of 3 nM [55]. DNAzymes have also been exploited for Ag^+^ detection [56]. As known, the most studied interaction between DNA and Ag^+^ is the specific binding with C residues [33,57,58]. This interaction was used to develop Ag^+^ biosensors [56,59,60] and for the assembling of fluorescent Ag nanoclusters [61,62]. Saran et al. [63] described the first Ag^+^-specific RNA-cleaving DNAzyme, successfully integrated in the specific biosensor. A catalytic beacon biosensor is obtained by labeling the 3′ end of the DNAzyme strand with a black hole, which, upon hybridization, quenches the signal of the fluorophore located on the 5′ end of the substrate. The Ag^+^-induced substrate cleavage enables fluorescence retrieval. A LOD of 24.9 nM was shown. 

Even though DNAzyme-based lead sensors generally demonstrate good sensitivity, the high synthesis cost of these molecule limited their extensive application. A DNA sensor based on Pb^2+^-stabilized G4 formation was proposed with a LOD of 3.79 ppb [64]. In the absence of Pb^2+^, a fluorescent tracer intercalates with the single-stranded coil and strongly emits. While, in the presence of Pb^2+^, the random-coil folds into a G4 structure leading to signal reduction (Figure 4a).

Commonly, a DNA-based biosensor for Pb^2+^ detection is frequently inclined to interference from Hg^2+^, due to the T–Hg^2+^–T interaction between Hg^2+^ and T residues. A label-free system with a LOD in the nanomolar range was optimized (also in the presence of Hg^2+^) based on the Pb^2+^-induced G4 formation with cationic polythiophene water-soluble conjugated polymer (PMNT), as described in the colorimetric transduction method section of this review [65]. 

In another arrangement, Y.F. Zhu et al. proposed a singly-labeled bifunctional probe consisting of a Cd^2+^-specific aptamer (CAP), capable to act as the recognition element for Cd^2+^ and the signal reporter [66]. The Cd^2+^ presence induces the switching of the CAP coil conformation to a stem-loop structure, which brings the four guanosine bases at the 5′ end close to 6-Fam at the 3′ end, resulting in fluorescence quenching. The biosensor showed a LOD of ~2 nM. 

Interestingly, G4 structures have been also exploited to develop a duplex functional fluorescent biosensor for distinct detection of Pb^2+^ and Hg^2+^ [67]. A K^+^-induced fluorescent G4 probe was assembled by a G-rich strand and a porphyrin. The sequence presents many T residues in addition to G residues, allowing to bind Pb^2+^ or Hg^2+^ selectively, changing into a more stably non-fluorescent G4 and a hairpin-like structure, respectively, resulting in PL reduction. LODs of 5.0 nM for Pb^2+^ and 18.6 nM for Hg^2+^ was reported.

As favorable as fluorescent nanomaterials, DNA-scaffolded silver nanoclusters (DNA–AgNCs) were successfully applied to a novel turn-on fluorescent biosensor [68]. When Pb^2+^ is present, the aptamer forms a G4 structure and the two darkish DNA/AgNCs positioned at the 3′ and 5′ terminus come closer, thus the fluorescence intensity increases [69]. A LOD as low as 3.0 nM was reported.

Light-up biosensors based on the target-induced release of fluorescence-labeled aptamer, from a complex with a Q-labeled short complementary sequence, were developed for Cd^2+^ and Pb^2+^ [70,71], with a LOD of 40 and 60.7 nM, respectively. 

A label-free aptasensor approach for Cd (II) detection was independently exploited by Y. Luan et al. [72] and B. Zhou et al. [73], combining an aptamer with unmodified dsDNA-specific dye. Based on the principle that hybridization of two aptamers boosts the fluorescence engendered during the reaction, B. Zhou et al. showed that, in the absence of Cd^2+^, SYBR green-I binds to the small groove of dsDNA (aptamer-complementary strand) establishing the dsDNA–dye complex and generating high fluorescence signal. The specific recognition and binding of aptamers with Cd^2+^ induce the release of the complementary strand from dsDNA and the aptamer conformational switching to a stem-loop structure, causing fluorescence decay (Figure 4d). A LOD of 0.34 ng/mL was reached.

Likewise, Y. Luan et al. reported that, induced by Cd^2+^ ions, the aptamer configuration changes from a random coil structure to an aptamer–Cd^2+^ complex. After the introduction of complementary strands and Pico Green dye (PG), a hybrid with the residual free aptamers that did not bind with Cd^2+^ is formed. This results in a higher PL signal (Figure 4e), allowing a higher sensitivity (LOD of 0.038 ng/mL) [72].

A comparable strategy was proposed for Pb^2+^ detection [74]. This biosensor is based on the principle that Pb^2+^ induces a structural change of G-rich thrombin aptamer from random coil to G4. This prevents its binding to the complementary sequences to form dsDNA and causes a fluorescence intensity decrease. The results showed a LOD of 1 ng/mL. 

A label-free fluorescence sensing system was also developed for As^3+^ detection by the exonuclease III (Exo III)-assisted cascade target recycling amplification process [75], exhibiting a LOD of 5 ng/L. As signal indicator and sensing element, the 2-amino-5,6,7-trimethyl-1,8-naphthyridine and the triple-helix molecular switch were used, respectively. This sensor could detect other HM ions with newly-designed triple-helix molecular switch by using aptamer sequences.

In the frame of functional device miniaturization, combining a microfluidic sample pre-treatment module (cation exchange resins) with a DNA aptamer immobilized photoluminescent graphene oxide QD (GOQD), a novel Pb^2+^ detection platform sensor was proposed [76], exhibiting a LOD of 0.64 nM. The DNA aptamer on the GOQD specifically captures the target (forming a G4 complex) which can trigger electron transfer from GOQD to Pb^2+^ upon UV irradiation, leading the GOQD PL quenching. 

#### 2.1.2. Electrochemiluminescence 

ECL is the process through which those intermediates generated at the electrodes undergo high-energy electron transfer reactions to produce an excited state that emits light, after relaxation to a lower level [77]; the process is initiated and modulated by switching an electrode voltage [78]. ECL allows small analyte detection at sub-picomolar concentration and wide dynamic range [79].

Various strategies were recently developed, such as biosensors that rely on the formation of the T–Hg^2+^–T and $\mathrm{Ru}{\left(\mathrm{phen}\right)}_{3}^{2+}$ or Ru-dppz, which permitted a LOD of 20 or 5.1 pM to be achieved, respectively [80,81]. 

In their study, X. Zhou et al. [82] reported that Bst DNA polymerase exhibits specific behaviors on the T–Hg^2+^–T biomimetic structure. The sensor exploits the MBs-labeled primer, planned to match the region of the circular padlock probe but with two T–T mismatches at the 3′ terminus. If Hg^2+^ is introduced, the DNA polymerase reaction with rolling circular amplification (RCA) mechanism is induced. Then, the resulting RCA products hybridize with the tris (bipyridine) ruthenium (TBR)-marked probes and sensed by ECL, once they are attracted to the magnet under the electrode. A LOD of 100 pM was shown.

One more method was designed by Meng Li et al., exploiting a Pb^2+^-specific DNAzyme, achieving a LOD of 9.6 × 10^−13^ M [83]. In this sensor, CdS QDs and DNAzyme with Ag/ZnO coupled structures were immobilized on agold nanodendrites-modified ITO electrode. Pb^2+^-activated DNAzyme moves the Ag/ZnO coupled structures near the surface to catalyze the reduction of part of the H_2_O_2_, inducing a signal intensity reduction.

Rather than utilizing DNAzyme, L. Lu et al. [84] proposed a sensor to detect Pb^2+^ using a graphene/AuNPs-modified electrode and ssDNA labeled with CdSe QDs. When Pb^2+^ is present, the G-rich ssDNA adopts the G4 conformation, leading to a shortening of the distance between the CdSe QDs and the graphene–AuNPs nanocomposite (Figure 5). This decreases the ECL intensity, allowing for the detection with a limit of 10^−10^ mol/L.

A novel ECL sensor to detect Pb^2+^ exploiting hemin/G4-based DNAzyme on the core-shell CdSe@CdS QDs, was proposed by X.-L. Du et al. [85]. Pb^2+^-induced G4 combines with hemin to form DNAzyme, which can catalyze H_2_O_2_ and oxidize 4-chloro-1-naphthol (4-CN) to form an insoluble precipitate. In the presence of Pb^2+^, more DNAzymes are produced and, thus, more 4-CN molecules are oxidized catalytically, leading to an output signal reduction. A LOD of 0.98 fM was achieved. 

Furthermore, a microfluidic paper-based device was successfully applied for concurrent detection of Pb^2+^ and Hg^2+^ based on the formation of G4 and T–Hg^2+^–T complexes, respectively [86]. Due to the different operational potentials of the two exploited labels (Si@CNCs and Ru@AuNPs), Pb^2+^ and Hg^2+^ can be quantified with a LOD of 10 pM and 0.2 nM, correspondingly. 

### 2.2. Colorimetric Method

In colorimetric sensors, the analyte detection occurs by means of a color change of the sensing element. Current technology based on colorimetry focuses on cost reduction, miniaturization, and in-situ detection. Generally, the recognition mechanism is based on molecular interaction on the substrate surface modified with NPs and functional groups [87]. 

For instance, DNA adsorption by citrate-capped AuNPs could be a function of DNA conformation. DNAs without stable secondary structures allow higher colloidal stability of AuNPs against salt-induced aggregation, because they are more efficiently adsorbed. A sensor exploiting Tl^+^-induced DNA folding and AuNPs was described by Hoang et al. [88]. The presence of Tl^+^ inhibits the DNA adsorption by AuNPs due to G4 sequence folding. Then, adding NaCl solution, a red-to-blue color change is observable because of NPs aggregation. A LOD of 4.6 μM was achieved.

Similarly, a specific Pb^2+^-induced G4 oligonucleotide (TBAA) probe and the cationic polythiophene (PMNT) readily form an electrostatic PMNT–TBAA red colored complex [65]. This sensor can detect Pb^2+^ traces at the micromolar level with the naked eye. Moreover, the authors report that, in the presence of Hg^2+^, the TBAA sequence (having adenine base) has a higher selectivity for Pb^2+^ than TBA (without adenine base in the sequence). As already reported, the same biosensor exhibits a lower LOD, when working in fluorometric mode.

In order to detect Hg^2+^, Zhu et al. [89] designed a sensor established on ssDNA, phthalic diglycol diacrylate (PDDA) and AuNPs. The T–Hg^2+^–T structure is much stronger than the interchain contact between ssDNA and PDDA. When the ssDNA recognizes Hg^2+^, a random coil-to-hairpin structure change occurs, avoiding ssDNA interaction with PDDA. Therefore, the free PDDA induces AuNP aggregation (Figure 6b), displaying a color change as a function of Hg^2+^ concentration. The LOD was as low as 5 nM.

A multianalyte responsive sensor, able to identify Ag^+^, Hg^2+^, Cr^3+^, Sn^4+^, Cd^2+^, Pb^2+^, Zn^2+^, and Mn^2+^ was designed by Tan et al. [90]. It is based on differential colorimetric and fluorescent response of FAM-DNA-AuNP once conjugated to a specific metal ion. A LOD of 50 nM was achieved. 

A different approach for visual detection of Hg^2+^, Ag^+^, Cu^2+^, Cd^2+^, Pb^2+^, Cr^6+^, and Ni^2+^ was reported by Hossain and Brennan [91]. An enzymatic reaction is optimized on a sol gel matrix-spotted bioactive paper device; β-galactosidase-substrate catalysis produces a colorimetric signal intensity, which is inversely proportional to the metal ion amount. The sensitivity was different for the diverse ions, as reported in Table 1. 

Another paper device was designed by J. Xu et al. [92], for the detection of Pb^2+^ via colorimetric and ECL techniques, exploiting a metal-specific DNAzyme and rGO–PdAu–GOx labeled oligonucleotide hybrid. The dual mode sensor showed a lower LOD in the ECL readout (0.14 nM) than in the colorimetric one (LOD: 1.6 nM).

A sensor based on mushroom apo-tyrosinase, entrapped in polyacrylamide gel, was developed by Kaur and Verma [93] in order to detect Cu^2+^, which act as enzyme cofactor for levodopa (L-DOPA) conversion, with a corresponding color change. The shown LOD was 0.01 ug/L.

A colorimetric Hg^2+^ detection was also optimized on a test strip, exploiting biotin-labeled and thiolated DNA-modified AuNPs and a T-rich DNA immobilized on the nitrocellulose membrane. Under optimized conditions, the LOD achieved for Hg^2+^ was 3 nM [94] or 5 nM [95]. 

Another colorimetric paper-based platform, involving T–Hg^2+^–T coordination chemistry and AuNPs aggregation, showed a LOD of 50 nM [96]. In a different way, a linker T-rich DNA and sequences complementary to the AuNPs DNA was designed to induce particle aggregation [97]. Remarkably, Hg^2+^ ions induce the linker DNA folding, allowing AuNPs to quickly disassemble and return to red color. A lower LOD (5.4 nM) was shown with respect to the AuNPs aggregation strategy described.

In another work concerning disposable lateral flow strips, the authors examined hairpin probe-modified AuNPs and T–Hg^2+^–T structure-based strategy. An additional T-rich, digoxin-labeled DNA strand was considered in order to hybridize with T–Hg^2+^–T coordination. Then, digoxin dsDNA–AuNPs complexes are captured by immunoreaction with the anti-digoxin Ab immobilized (Figure 6a) on the strip and revealed by a red band [98]. Interestingly, a lower LOD for Hg^2+^ was shown (0.1 nM), when compared with non-immunochromatographic approaches.

On parallel route, an Exo III-catalyzed target recycling approach was employed to improve the sensitivity of a similar disposable strip on the basis of Hg^2+^-triggered toehold binding. Using AuNPs as the tracer enables the detection of Hg^2+^ with a LOD of ~1 pM [99]. Moreover, in order to sense Cu^2+^, a lateral flow device based on specific ion-dependent DNA-cleaving DNAzyme and AuNPs was developed, achieving a LOD of 10 nM [100].

### 2.3. Evanescent Wave 

This method employs the evanescent field of an optical fiber to excite the biological recognition molecule, producing a fluorescence signal. An optical fiber is essentially a cylindrical dielectric waveguide with an inner core having a refractive index greater than that of the cladding. EW exploits the phenomenon of total internal reflection (i.e., the transition of light in the optical fiber by continually reflecting off the cladding–core interface without data loss. If the cladding is removed, the evanescent field can interact with the fiber surroundings [101]. In order to immobilize biological recognition elements on the optical fiber surface, various methods have been reported [102], such as direct or mediated covalent immobilization, adsorption, or entrapment in polymer matrices.

Based on this transduction, a DNAzyme-based sensor for Pb^2+^ detection was developed by N. Yildirim et al. [103], using GR-5 Pb^2+^-dependent DNAzyme. In the presence on Pb^2+^, the active molecule can catalyze the cleavage of an RNA base embedded in the fluorescent-labeled (Cy5.5) DNA substrate. After that, the released Cy5.5-labeled fragments hybridize with the complementary strands immobilized on the optical fiber, and Pb^2+^ detection is revealed by PL signal. Restored over 100 times, this sensor showed no important performance decay and a LOD of 1.03 nM.

Another sensor based on DNAzyme for Pb^2+^ detection was realized by R. Wang et al. [104] The whole sensing procedure requires three steps: (i) Pb^2+^ ion determines the cleavage of the DNA substrate at the single RNA site, by the DNAzyme, causing the release of a short ssDNA arm which will be used in the second step; (ii) the released ssDNA hybridizes with a complementary DNA strand immobilized on MBs in solution, causing the competitive detachment of the originally hybridized probes (streptavidin–ssDNA–Cy5.5) and the remaining dsDNA–MBs complex is removed by magnetic separation; (iii) the released signal probe is pumped into the flow cell of the biosensing platform, where it can be captured by the desthiobiotin-modified fiber. A LOD of 1 nM was achieved, with the possibility to be reused at least 250 times.

To avoid the use of MBs and to keep the same performance, a similar system was optimized for Hg^2+^ sensing [105], by introducing a quencher. Fluorescein-labeled DNA strands with streptavidin (DNA–SA) were designed to hybridize with Q-labeled cDNA strands (Q–DNA). Hg^2+^ induces an enhancement in the PL signal because of the Q-DNA release, once the DNA-SA is bound to form a hairpin structure stabilized by the T–T mismatch (Figure 7a). A LOD of 1.06 nM was shown. 

With a similar LOD, F. Long et al. [106] developed another sensor based on T–T mismatch pairs and fluorescently (Cy5.5)-labeled cDNA. The DNA probe has two functional elements: a T–T mismatch pair that can bind with Hg^2+^ ions, and a short sequence that can hybridize with the fluorescently-labeled cDNA. Via a structure competitive mode, Hg^2+^ ions lead to a decrease of the signal. The authors stated a LOD of 2.1 nM, with a reproducibility over 100 times.

In more recent works, the same author proposed two structure-switching DNA optical biosensors for detection of HM ions [107,108]. The developed approaches for Hg^2+^ or Pb^2+^ detection, respectively, differ by the FNA-based strategy exploited (i.e., T–T mismatch or G4 aptamer (Figure 7b)).

Once introduced in the modified optofluidic cell, the specific metal ion-induced aptamer conformation change reduces the binding of a fluorescently-labeled free DNA with the immobilized DNA probe, causing a decrease of fluorescence signal. A LODs of 1.2 nM for Hg^2+^ and 0.22 nM for Pb^2+^ were reported. One more sensor for Hg^2+^ and Pb^2+^ detection, based on T–T mismatch-containing DNA or DNAzyme, respectively, was developed by S. Han et al. [109]. In this system, the detection of HM contaminants is carried out exploiting two complementary DNA sequences, one labeled with a Cy3.3 and one with a Q. The metal ion induces structural modification, causing paired-strand dehybridization and, consequently, the binding of the Cy3.3-labeled segment to the cDNA probe on the fiber surface. By excitation via EW, a detectable fluorescence signal is generated, with a LOD of 22 pM for Hg^2+^ and 20 nM for Pb^2+^.

### 2.4. Surface-Enhanced Raman Spectroscopy 

In SERS, definite metallic surfaces are used to intensify Raman scattering of the specific element, by benefitting from localized surface plasmon resonances. Noteworthy: (i) SERS spectra can provide information about the chemical structure of the target, (ii) it permits rapid detection; (iii) weak Raman scattering of water makes its background signal negligible [110]. 

A highly sensitive DNAzyme-centered SERS quadratic amplification method, based on a bio-barcode and hybridization chain reaction, was developed for Pb^2+^ detection [111]. The system includes a DNAzyme-MB complex, a SERS active bio-barcode (composed of the capture probe matching the stem of hairpin DNA, and Raman dye-labeled DNA) on top of AuNPs, to produce a strong SERS signal. Adding Pb^2+^, once a DNA–Pb^2+^ complex is formed, a catalytic cleavage of the substrate sequence takes place, giving rise to a series of reaction steps, finally leading to quantitative Pb^2+^ detection with a LOD of 70 fM. The method can be further applied to different elements by substituting the lead-responsive DNAzyme with the specific functional DNA.

Combining a specific As^3+^ aptamer, a reporter molecule and Raman-labeled Au@Ag core–shell NPs, a novel SERS strategy was proposed [112]. In the absence of As^3+^ ions, the aptamer and the reporter are absorbed on Au@Ag; while when they are present, the As^3+^ ions compete with NPs for binding to the aptamer, inducing its release from the NP surface, which then aggregate generating SERS “hot spots”. This amplification strategy allowed to obtain a LOD of 0.1 ppb.

Likewise, a label-free SERS device was developed for sensing of Hg^2+^ [113], exploiting aptamer-derivatized SiO_2_@Au core–shell NPs. The DNA aptamer consists of two segments, one containing guanine (G) and adenine (A) bases as signal reporters and the other segment, with consecutive T, as the Hg^2+^ recognition element. The single strand poly–T shows a flexible structure; when present, Hg^2+^ ions cause the formation of T–Hg^2+^–T pairs via N–Hg^2+^–N J-coupling bonding. As a result, the DNA molecule adopts vertical alignment (Figure 8a), changing respective Raman intensities of A and G bases in the sequence. In this system, Hg^2+^ detection showed a limit of 10 nM.

The system formerly suggested by L. Zhang et al., requiring a fluorescent label, resulted as more sensitive [114]. It was based on nanoporous gold as the plasmonic surface and a Cy5-labeled aptamer as the optical tag for Hg^2+^ detection. The coordination of a pair of poly–T oligos with the metal ion induces the molecule perpendicular arrangement, as above described. Consequently, an amplification of the fluorophore surface-enhanced resonance Raman scattering signal (SERRS) variation is observed. A LOD of 1 pM was reported.

Exploiting the same Hg^2+^ biorecognition element, W. Ma et al. obtained SERS-enhanced Hg^2+^ detection, thanks to the T–Hg^2+^–T-induced assembly of gold nanostar dimers [115]. A great number of “hotspots” were formed, inducing an increase of electromagnetic field over an extensive connecting region. A LOD of 0.8 pg/mL was reached, showing a higher sensitivity if compared with the similar strategy exploited for As^3+^ detection (Figure 8b) described in [112].

A selective single nanowire-on-film (SNOF) sensor for Hg^2+^ was realized exploiting the hybridization between T-rich ssDNAs and complementary Cy5-labeled DNAs [116]. In the presence of Hg^2+^, T-rich DNAs fold into hairpin structures to form T–Hg^2+^–T pairs, leading to an easy release of Cy5-tagged DNAs. The free-labeled ssDNAs are then caught by the SNOF derivatized with cDNAs, turning on the SERRS signal. A LOD of 100 pM was achieved. 

### 2.5. Förster Resonance Energy Transfer 

FRET is a physical process where a non-radiative energy transfer from an excited state molecule (donor) to another molecule (acceptor) occurs, by means of intermolecular long-range dipole–dipole coupling. When the energy transfer takes place from donor to acceptor, the fluorescence intensity of the donor decreases. An essential requirement for effective transfer is that an overlap exists between the fluorescence spectrum of the donor and the absorbance spectrum of the acceptor. The rate and the efficiency of the energy transfer depends on the sixth power of the distance between donor and acceptor [117]. Various combinations of donor–acceptor pairs have been used, such as two fluorophores, fluorophore with AuNP, fluorophore with an intercalator or with a dark absorber [118].

For instance, T–Hg^2+^–T complex-induced conformational change of ssDNA allows one-step sensing of Hg^2+^ in a AuNPs-based sensor developed by G. Wang et al. [119]. The AuNPs were used as acceptor and FAM as donor. The DNA probes tagged with a FAM on 3′ and thiol on 5′ end were bound to AuNPs. In order to enable an enhanced FRET process, FAM and AuNPs need to be close to each other, as occurs when the conformation of the DNA probe changes into a hairpin structure leading to fluorescence signal quenching. A LOD of 8 nM was achieved with this approach.

Using the catalyzed hairpin assembly technique, a different aptasensor for Hg^2+^ was developed by K. Chu-mong et al. [120]. The suggested strategy exploits a Hg^2+^ aptamer–catalyst complex and two hairpin DNA: H1—fluorescein (donor) and H2—tetramethylrhodamine (acceptor). The formation of the T–Hg^2+^–T complex releases the catalyst strand, triggering the signal amplification step: Hairpin assembly is catalyzed turning H1 and H2 into a duplex. Consequently, FRET efficiency increases and the Hg^2+^ concentration can be measured with nanomolar LOD. 

An opposite functional scheme was described for Ag^+^ sensing by Y.-J. Chen et al. [121]. Fusing the cyan fluorescent protein (donor) and the yellow fluorescent protein through a truncated CupR protein. CupR contains a dimerization helix and a metal binding domain. The presence of Ag^+^ ions causes the decrease in FRET efficiency by inducing conformational change of the biorecognition element (Figure 9b).

More complex systems were also designed to simultaneously detect several HMs. Using the establishment of C–Ag^+^–C and T–Hg^2+^–T complexes, Cy5 and TAMRA as acceptors and CdTe QDs as donors, C. Hao et al. [122] successfully detected Ag^+^ and Hg^2+^ with a LOD of 2.5 and 1.8 nM, respectively. When a specific ion is present, if donor and acceptor are in close proximity, a fluorescence intensity increase will take place.

Interestingly, J. Xia et al. engineered specific DNA sequences for Hg^2+^, Pb^2+^, and Ag^+^, integrating them in two DNA strands and labeling these strands with multicolor fluorophores, in order to realize a cascade FRET [123]. In this way, only one excitation wavelength is needed to obtain a fingerprint-like spectrum in multianalyte monitoring. The sensor works in a dynamic range from 100 nM to 2 μM for Ag^+^ and Hg^2+^ and can detect as low as 20 nM Pb^2+^.

As already described, M. Hoang et al. [88] demonstrated that a sensor based on G4 DNAs, FAM (donor), and TMR (acceptor) can be used for Tl^+^ detection (Figure 9a) with a LOD of 59 μM, unusually lower than that showed by the colorimetric transduction method.

### 2.6. Surface Plasmon Resonance 

When light incides on a metal surface, plasmons are generated, whose propagation is very sensitive to the variations in the material refractive index. This alteration can be caused by biomolecular interaction (probe–target) or by a structural modification of the molecules linked to the sensor surface [124,125]. 

For instance, the detection of Cu^2+^ was achieved by associating a SPR biosensor with the competitive adsorption of proteins [126]. The interaction between bio-receptors (native proteins (albumin)) and Cu^2+^ ions leads to protein denaturation, inducing a lower affinity between protein–gold surface, thus initiating the competitive displacement by the native one (Figure 10a), which is monitored by SPR measurement with a LOD down to 0.1 mg/L.

A mercury (II) sensor, based on the dissociation rate of the trans-acting factor MerR from the cis-element, was investigated by SPR [128]. The sensor, modified with dsDNA including the cis-element (Pmer), can monitor the dissociation stage of MerR or protein-tagged MerR from the cis-element, enabling measurement of Hg^2+^ with a LOD of 5 µg/L.

Non-specific adsorption can influence the SPR accuracy. In this direction, a laser scanning confocal imaging and SPR were combined to realize a system for Hg^2+^ detection [127]. By adding Hg^2+^, the rhodamine-labeled ssDNA folds into the T–Hg^2+^–T-mediated hairpin structure and this structural change attracts the rhodamine fraction in proximity to the Au surface (Figure 10b). A double effect is observed: SPR signal heightening and PL quenching. From the PL quenching status, the strand folding is monitored in real time, and the Hg^2+^ detection is recorded by the SPR signal, as a function of refractive index and thickness variations of the Au surface, achieving a LOD of 0.01 ng/mL.

In this rich context, the summary of the recently described biosensors is schematized in Table 1. Here, biosensors are classified by their sensitivity (from lower to higher LOD) with respect to a specific ion, within the same transduction method on real samples, but with diverse bio-signaling strategies. 

**Figure 11:**
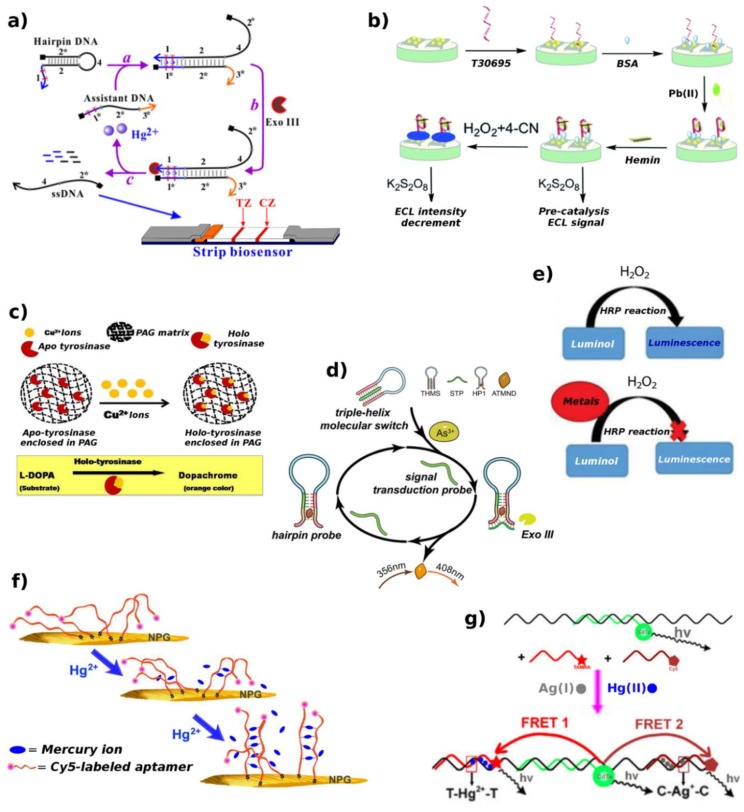
Most sensitive biorecognition strategy/specific HM ion, in real samples. (**a**) Disposable strip biosensor based on Hg^2+^-induced toehold binding and Exo III-assisted signal amplification [99]; (**b**) ECL Pb^2+^ sensor based on hemin/G4-based DNAzyme biocatalysis [85]; (**c**) Cu^2+^ triggered conversion of apo-tyrosinase disc into holo-tyrosinase one, and consequent L-DOPA to dopachrome transformation [93]; (**d**) As^3+^ detection by Exo III-assisted cascade target-recycling amplification scheme [75]; (**e**) Possible on-site analysis of HMs by means of the HRP-based bioassay [40]; (**f**) Aptamer-modified NPG-based SERRS sensing of Hg^2+^ [114]; (**g**) Ag+ and Hg^2+^ detection by FRET between QD and organic dyes [122]. Adapted with permission of the Publishers.

Moreover, in order to clearly illustrate the most sensitive recent methods as well as the bio-recognition elements giving the lowest detection limits, two comparative tables (Table 2 and Table 3, respectively) are proposed and shown below. Then, a representative drawing (Figure 11) aims to show the most sensitive detection strategies, with respect to a specific analyte, applied in real samples.

## 3. Conclusions

Nucleic acids, biocatalysts, antibodies, receptors, etc., are natural or biomimetic elements with distinctive features such that they have been engaged as recognition probes since the first public biosensor description in a paper, over 55 years ago, in which Dr. L. C. Clark termed his device as an “enzyme electrode” [129]. In the fields of environmental and food analysis, water and milk exemplify the matrices involved in potential HM ion contamination. In this context, although most of the developed systems were tested only on buffered solutions, plenty of optical biosensors appropriate for real samples showed up in the last decade for possible environmental and food quality monitoring applications. Continuous advances are presented, exploiting nano-microtechnology and biotechnology, such as for miniaturization of integrated systems, genetic engineering of receptors, enzymes, and microorganisms, as well upgrading of bioelement immobilization methods.

Thus far, a number of metals can be selectively sensed by DNA sequences down to the low ppb level [11]. Accordingly, Table 3 shows that direct metal binding DNA sequences allow obtainment of the highest sensitivity. In detail, the biorecognition mechanisms more frequently adopted are those based on T–T mismatch and G-quadruplex, respectively, for Hg^2+^ and Pb^2+^; nonetheless, to the same extent, functional nucleic acids (DNAzyme) are exploited for Pb^2+^. Among the optical biosensors here reviewed, those applied in real samples, namely milk and water (specifically tap water, mineral water, surface water, underground water), have been assessed by spike test, largely for Hg^2+^, Pb^2+^, and Cd^2+^ ions, in descending order, and, in small part, also for Cu^2+^, Ag^+^, Cr^3+^, As^3+^, Tl^+^, and Sn^4+^ ions, as summarized in Table 1. Remarkably, ten multianalyte optical devices (able to sense up to eight HM ions) were shown out of a total of more than seventy biosensors here considered, with a large part of them designed to quantitatively discriminate between two ions.

## Figures and Tables

**Table 1:** Recently reported biosensors sorted in function of the lower limit of detection (LOD), with respect to a specific analyte within the specific transduction method. Cited literature refers to analytic procedures performed on real samples.

Transduction Method	Analyte	Receptor	LOD	Linear Range	Real Sample	Reference
**Luminescence**	Hg^2+^	Enzyme	1 pg/mL	(5–500) pg/mL	T.W., M.W.	[41]
**Luminescence**	Hg^2+^	DMB-DNA (T–Hg^2+^–T)	16 pM	(6.2 × 10^−10^–1.2 × 10^−8^) M (1.2 × 10^−8^–1.2 × 10^−6^) M	T.W., S.W.	[38]
**Luminescence**	Hg^2+^	DMB-DNA (T–Hg^2+^–T)	3.5 × 10^−10^ M	(1.0 × 10^−9^–1.5 × 10^−7^) M	S.W.	[39]
**Luminescence**	Hg^2+^	Enzyme	1 μg/L	(1–60) μg/L	S.W.	[40]
**Luminescence**	Pb^2+^	Enzyme	0.7 μg/L	(0.7–54) μg/L	S.W.	[40]
**Luminescence**	Cd^2+^	Enzyme	0.02 μg/L	(0.02–45) μg/L	S.W.	[40]
**Fluorescence**	Pb^2+^	DMB-DNA (G4)	0.64 nM	---	T.W., S.W.	[76]
**Fluorescence**	Pb^2+^	L-DNAzyme	3 nM	(5–100) nM	S.W.	[55]
**Fluorescence**	Pb^2+^	DMB-DNA (G4)	3.0 nM	(5–50) nM	T.W., S.W.	[68]
**Fluorescence**	Pb^2+^	DMB-DNA (G4)	1 ng m/L	1 ng m/L to over 1 mg m/L	T.W., M.W.	[74]
**Fluorescence**	Pb^2+^	DMB-DNA (G4)	5.0 nM	(10–200) nM	S.W.	[67]
**Fluorescence**	Pb^2+^	DMB-DNA (G4)	6 nM	(0–120) nM	T.W.	[65]
**Fluorescence**	Pb^2+^	DNAzyme		---	S.W.	[53]
**Fluorescence**	Pb^2+^	DMB-DNA	60.7 nM	(100–1000) nM	S.W.	[71]
**Fluorescence**	Hg^2+^	DMB-DNA (T–Hg^2+^–T; G4)	0.17 nM	(0.5–50) nM	T.W.	[50]
**Fluorescence**	Hg^2+^	DMB-DNA (T–Hg^2+^–T)	0.2 nM	(2–160) nM	S.W.	[52]
**Fluorescence**	Hg^2+^	DMB-DNA (T–Hg^2+^–T)	3.6 nM (turn-off) 8.6 nM (turn-on)	(0.01–10) μM ---	M.W., milk.	[51]
**Fluorescence**	Hg^2+^	Enzyme	<10 nM	---	T.W., S.W.	[47]
**Fluorescence**	Hg^2+^	DMB-DNA (G4)	18.6 nM	(200–1000) nM	S.W.	[67]
**Fluorescence**	Hg^2+^	DMB-DNA (T–Hg^2+^–T)	48 nM	(0–0.5) μM (0.5–6) μM	S.W.	[49]
**Fluorescence**	Cd^2+^	DMB-DNA	0.038 ng/mL	(0.10–100) μg/mL	S.W.	[72]
**Fluorescence**	Cd^2+^	DMB-DNA	2.15 nM	(7.19–100) nM and 200 nM–5.0 μM	T.W., S.W., U.W.	[66]
**Fluorescence**	Cd^2+^	DMB-DNA	0.34 μg/L	(1.12–224.82) μg/L	S.W., T.W., U.W.	[73]
**Fluorescence**	Cd^2+^	DMB-DNA	40 nM	(0–1000) nM	S.W.	[70]
**Fluorescence**	Cd^2+^	Enzyme	50 µM	---	T.W., S.W.	[47]
**Fluorescence**	Ag^+^	RNA-cleaving DNAzyme	24.9 nM	---	S.W.	[63]
**Fluorescence**	Ag^+^	DMB-DNA (C–Ag^+^–C)	0.31 μM	(0–10) μM	S.W.	[49]
**Fluorescence**	As^3+^	DMB-DNA	5 ng/L	10 ng/L–10 mg/L	T.W., S.W.	[75]
**Fluorescence**	Cu^2+^	Enzyme	0.176 ng/mL	(0–2.4) ng/mL	S.W.	[48]
**Fluorescence**	Cu^2+^	DNAzyme	0.6 ppb	---	S.W.	[53]
**Fluorescence**	Cu^2+^	Enzyme	---	---	T.W., S.W.	[47]
**ECL**	Hg^2+^	DMB-DNA (T–Hg^2+^–T)	5.1 pM	(5.0 × 10^−11^–1.0 × 10^−8^) M	T.W., S.W.	[81]
**ECL**	Hg^2+^	DMB-DNA (T–Hg^2+^–T) and DNA polymerase	100 pM	---	T.W., S.W.	[82]
**ECL**	Hg^2+^	DMB-DNA (T–Hg^2+^–T; G4)	0.2 nM	(5.0 × 10^−10^–1.0 × 10^−6^) M	S.W.	[86]
**ECL**	Pb^2+^	G4 based DNAzyme	0.98 fM	(1.0 × 10^−15^–1.0 × 10^−11^) M	S.W.	[85]
**ECL**	Pb^2+^	DNAzyme	9.6 × 10^−13^ M	(5.0 × 10^−12^–4.0 × 10^−6^) M	S.W.	[83]
**ECL**	Pb^2+^	DMB-DNA (C–Pb^2+^–C; G4)	10 pM	(3 × 10^−11^–1.0 × 10^−6^) M	S.W.	[86]
**ECL**	Pb^2+^	DMB-DNA (G4)	10^−10^ mol/L	(10^−8^–10^−10^) mol/L	S.W.	[84]
**ECL**	Pb^2+^	DNAzyme; Enzyme	0.14 nM	(0.5–2000) nM	T.W., S.W.	[92]
**Colorimetry**	Pb^2+^	DNAzyme	1.6 nM	(5–2000) nM	T.W., S.W.	[92]
**Colorimetry**	Pb^2+^	DMB-DNA (G4)	---	---	T.W.	[65]
**Colorimetry**	Cu^2+^	Enzyme	0.01 μg/L	(0.1–25) μg/L	M.W., milk	[93]
**Colorimetry**	Hg^2+^	DMB-DNA (T–Hg^2+^–T); Enzyme	1 pM	---	S.W.	[99]
**Colorimetry**	Hg^2+^	DMB-DNA (T–Hg^2+^–T); Ab*	0.1 nM	(0.1–100) nM	S.W.	[98]
**Colorimetry**	Hg^2+^	DMB-DNA (T–Hg^2+^–T)	0.15 nM (UV-vis spectroscopy) 5 nM (naked eye)	(0.25–500) nM (UV-vis spectroscopy)	S.W.	[89]
**Colorimetry**	Hg^2+^	DMB-DNA (T–Hg^2+^–T)	3 nM	---	S.W.	[94]
**Colorimetry**	Hg^2+^ Ag^+^ Cu^2+^ Cd^2+^ Pb^2+^ Cr^6+^ Ni^2+^	Enzyme	0.001 ppm 0.002 ppm 0.020 ppm 0.020 ppm 0.140 ppm 0.150 ppm 0.230 ppm	---	T.W., S.W.	[91]
**Colorimetry**	Hg^2+^	DMB-DNA (T–Hg^2+^–T)	5.4 nM	(0–1500) nM	S.W.	[97]
**Colorimetry**	Hg^2+^	DMB-DNA (T–Hg^2+^–T)	50 nM	---	S.W.	[96]
**Colorimetry**	Tl^+^	DMB-DNA (G4)	4.6 μM	---	S.W.	[88]
**Colorimetry**	Ag^+^ Hg^2+^ Cr^3+^ Sn^4+^ Cd^2+^ Pb^2+^ Zn^2+^ Mn^2+^	DMB-DNA	~50 nM	---	S.W.	[90]
**EW**	Pb^2+^	DMB-DNA	0.22 nM	(1.0–300) nM	M.W., T.W.	[108]
**EW**	Pb^2+^	DNAzyme; Protein	1 nM	(20–800) nM	T.W., M.W.	[104]
**EW**	Pb^2+^	DNAzyme	1.03 nM	---	T.W.	[103]
**EW**	Pb^2+^	DNAzyme	20 nM	---	E.W.	[109]
**EW**	Hg^2+^	DMB-DNA (T–Hg^2+^–T)	22 pM	22 pM–10 nM	E.W.	[109]
**EW**	Hg^2+^	DMB-DNA (T–Hg^2+^–T); Protein	1.06 nM	(75–1000) nM	S.W., M.W., T.W.	[105]
**EW**	Hg^2+^	DMB-DNA (T–Hg^2+^–T); cDNA	2.1 nM	---	M.W., T.W.	[106]
**SERS**	As^3+^	DMB-DNA	0.1 ppb	(0.5–10) ppb	S.W.	[112]
**SERS**	Pb^2+^	DNAzyme	70 fM	0.1 pM–0.1 μM	T.W., R.W.	[111]
**SERRS**	Hg^2+^	DMB-DNA	1 pM	---	U.W.	[114]
**SERS**	Hg^2+^	DMB-DNA (T–Hg^2+^–T)	0.8 pg/mL	(0.002–1) ng m/L	T.W.	[115]
**SERS**	Hg^2+^	DMB-DNA (T–Hg^2+^–T)	10 nM	(1 × 10^−8^–1 × 10^−3^) M	U.W., S.W.	[113]
**FRET**	Hg^2+^	DMB-DNA (T–Hg^2+^–T)	1.8 nM	---	T.W., L.W.	[122]
**FRET**	Hg^2+^	DMB-DNA (T–Hg^2+^–T)	(7.03 ± 0.18) nM	(10.0–200.0) nM	S.W.	[120]
**FRET**	Hg^2+^	DMB-DNA (T–Hg^2+^–T)	8 nM	(20–90) nM	T.W.	[119]
**FRET**	Ag^+^	DMB-DNA (C–Ag^+^–C)	2.5 nM	---	T.W., S.W.	[122]
**FRET**	Ag^+^	Protein	---	---	T.W., S.W.	[121]
**FRET**	Tl^+^	DMB-DNA (G4)	59 μM	---	S.W.	[88]
**LSCI-SPR**	Hg^2+^	DMB-DNA (T–Hg^2+^–T)	0.01 ng/mL	(0.01–100) ng/mL	T.W.	[127]
**SPR**	Hg^2+^	DMB-DNA	5 μg/L	(10^1^–10^4^) μg/L	M.W.	[128]
**SPR**	Cu^2+^	Protein	~0.1 mg/L	---	T.W., M.W.	[126]

Tap water (T.W.); mineral water (M.W.); surface water (S.W.); underground water (U.W.); environmental water (E.W.). Direct metal binding DNA sequence (DMB-DNA); Antibody (Ab*) (*indirectly exploited).

**Table 2:** Most sensitive transduction methods, with respect to the specific analyte (n. of published works ≥ 2), applied in real samples.

Analyte	Transduction Method	LOD	Reference
Pb^2+^	ECL	0.98 fM	[85]
Hg^2+^	SERRS	1 pM	[114]
Hg^2+^	Colorimetry	1 pM	[99]
Cd^2+^	Luminescence	0.02 μg/L	[40]
As^3+^	Fluorescence	5 ng/L	[75]
Cu^2+^	Colorimetry	0.01 μg/L	[93]
Ag^+^	FRET	2.5 nM	[122]

**Table 3:** Bio-recognition elements giving the lowest detection limits, within the same transduction method, with respect to a specific analyte (n. of published works ≥ 3).

Transduction Method	Analyte	Signaling Strategy	LOD	Reference
**Luminescence**	Hg^2+^	Enzyme	1 pg/mL	[41]
**Fluorescence**	Pb^2+^	G4 aptamer–GOQD	0.64 nM	[76]
**Fluorescence**	Hg^2+^	AO–DNA Aptamer (T–Hg^2+^–T; G4)	0.17 nM	[50]
**Fluorescence**	Cd^2+^	Aptamer/cDNA	0.038 ng/mL	[72]
**Fluorescence**	Cu^2+^	Alcohol Oxidase inhibition	0.176 ng/mL	[48]
**ECL**	Pb^2+^	Hemin/G4-based DNAzyme	0.98 fM	[85]
**ECL**	Hg^2+^	Hg^2+^-specific AuNP–ssDNA/cDNA	5.1 pM	[81]
**Colorimetric**	Hg^2+^	Hairpin DNA(T–Hg^2+^–T)/Exonuclease III	1 pM	[99]
**Colorimetric**	Pb^2+^	DNAzyme/GO–PdAu–(GOx)–ssDNA	1.6 nM	[92]
**EW**	Pb^2+^	Cy5.5–G4 aptamer	0.22 nM	[108]
**EW**	Hg^2+^	Quencher T-rich DNA/ Cy3–cDNA/ssDNA probe (T–Hg^2+^–T)	22 pM	[109]
**SERRS**	Hg^2+^	Cy5–Aptamer–NPG (T–Hg^2+^–T)	1 pM	[114]
**FRET**	Hg^2+^	TAMRA–ssDNA/QD–ssDNA (T–Hg^2+^–T)	1.8 nM	[122]
